# *rx*COV is a quantitative metric for assessing immunoassay analyte fidelity

**DOI:** 10.1038/s41598-022-27309-1

**Published:** 2023-01-03

**Authors:** Rhonda M. Brand, Danielle Pitlor, E. Jeffrey Metter, Beth Dudley, Eve Karloski, Ashley Zyhowski, Randall E. Brand, Shikhar Uttam

**Affiliations:** 1 Department of Medicine, University of Pittsburgh, Pittsburgh, PA USA; 2 Magee Women’s Research Institute, University of Pittsburgh, Pittsburgh, PA USA; 3 Department of Bioengineering, University of Pittsburgh, Pittsburgh, PA USA; 4 Department of Neurology, University of Tennessee, Memphis, TN USA; 5 UPMC Hillman Cancer Center, University of Pittsburgh, Pittsburgh, PA USA; 6 Department of Computational and Systems Biology, University of Pittsburgh, Pittsburgh, PA USA

**Keywords:** Immunology, Chemokines, Cytokines, Translational immunology, Diagnostic markers, Predictive markers, Prognostic markers, Gastrointestinal cancer

## Abstract

Immunoassay based bioanalytical measurements are widely used in a variety of biomedical research and clinical settings. In these settings they are assumed to faithfully represent the experimental conditions being tested and the sample groups being compared. Although significant technical advances have been made in improving sensitivity and quality of the measurements, currently no metrics exist that objectively quantify the fidelity of the measured analytes with respect to noise associated with the specific assay. Here we introduce *ratio of cross*-coefficient-of-variation (*rx*COV), a fidelity metric for objectively assessing immunoassay analyte measurement quality when comparing its differential expression between different sample groups or experimental conditions. We derive the metric from first principles and establish its feasibility and applicability using simulated and experimental data. We show that *rx*COV assesses fidelity independent of statistical significance, and importantly, identifies when latter is meaningful. We also discuss its importance in the context of averaging experimental replicates for increasing signal to noise ratio. Finally, we demonstrate its application in a Lynch Syndrome case study. We conclude by discussing its applicability to multiplexed immunoassays, other biosensing assays, and to paired and unpaired data. We anticipate *rx*COV to be adopted as a simple and easy-to-use fidelity metric for performing robust and reproducible biomedical research.

## Introduction

Biosensing assays such as antibody based enzyme-linked immunoabsorbent assay (ELISA) and its multiplexed counterpart (mELISA), are ubiquitously used methods that utilize antigen-antibody binding to determine with high sensitivity, concentration of analytes in a wide variety of bioanalytical settings^1^. Their ease-of-use coupled with improvements in measurement technology and quality control^2,3^ has made these immunoassays a mainstay in diverse biomedical research and its clinical applications^4,5^. More recently, nucleic acid-based probes, such as aptamers have also been developed^6,7^. Aptamers are short single-stranded RNA or DNA sequences that can replace antibodies in ELISA to bind targets of interest with improved specificity and affinity^6–8^. The resulting enzyme-linked aptamer assay (ELAA) has the potential to provide fast, low-cost, and improved target detection alternatives to ELISA^8^. However, measurement of target expression in these biosensing assays is fundamentally confounded by intrinsic biological heterogeneity, variations in preanalytical variables between different sample groups, variablity is user-dependent assay implementation, and imperfection of the assay itself due to varying degrees of target-dependent cross-reactivity and affinity^9–12^. These confounding factors together combine to generate stochastic noise that we refer to as assay-associated noise. Despite improvements both in the quality and biochemistry of biosensing technologies, assay-associated noise continues to confound analyte measurements. This noise is especially important to account for when comparing sample groups or experimental conditions with analyte expression in low signal to noise regime, or when differences in expression between the comparison groups are small. In such scenarios comparison of analyte expression between the two groups in the background of assay-associated noise can falsely suggest significance, or conversely the lack of it. Therefore, it is essential to establish the validity of differential analyte expression with respect to assay-associated noise, which we refer to as analyte fidelity. Establishing analyte fidelity is necessary for subsequent analyses to be meaningful.

Currently, protocol-level experimental techniques are used to ameliorate these assay-associated noise effects by carefully implementing best practices in assay design^3^. Additionally, repeat measurements can be performed to average out noise effects and improve the analyte signal to noise ratio (SNR). However, currently no quantitative metric exists to ascertain if the SNR has sufficiently improved, or how many experimental replicates are sufficient for each individual experiment to ensure good SNR. As a result, researchers use an ad-hoc number, usually justified based on logistics, cost or accepted practice. This can lead to inconsistent findings and reduced translational efficacy, particularly in research where reproducibility is a key criterion such as in biomarker discovery and drug development. Furthermore, in mechanism-focused basic research, lack of analyte fidelity can validate incorrect hypotheses resulting in spurious conclusions.

To overcome these challenges, we have developed a simple and easy-to-use quantitative metric that objectively characterizes analyte fidelity when comparing different sample groups or treatment conditions in individual experiments. As a result, it is also capable of identifying spurious statistical significance. We have developed the metric from first principles and show that it naturally emerges as a ratio of two modified forms of coefficient-of-variation (COV) statistic (see Eq. 2c). We, therefore, refer to it as *ratio of cross*-Coefficient-of-Variation (*rx*COV). COV itself has been used as a unit-free interpretable measure of precision in assessing reliability and repeatibility of collected data in a range of disciplines including biology and medicine^13–15^. The two modified COV terms together quantify the relative effect size of the differential analyte expression with respect to the assay-associated noise. We show that this relative effect size objectively characterizes analyte fidelity. Furthermore, we demonstrate that *rx*COV can determine whether the number of experimental replicates is sufficient to ensure good SNR and prevent spurious findings. Finally, as the fidelity metric is computed separately for each analyte and for any two sets of sample groups or condtions, it can be easily computed in parallel for multiple individual analytes in a multiplexed setting. It may also be extended to measure analyte fidelity between multiple different sample groups or experimental conditions.

## Results

### Fidelity metric: *rx*COV

We derive *rx*COV via a two step process. We first define a function that quantifies relative change between the differential analyte expression and the measurement noise associated with the assay. We next use this function to derive the fidelity metric.

#### Function of relative change

Let random variables *X* and *Y* respectively represent the expressions of an analyte of interest in samples belonging to two different patient groups or experimental conditions being compared. And let $X^{\prime }$ and $Y^{\prime }$ represent their repeat measurements on aliquots drawn from the same samples. The difference between the two sets of measurements on the same sample, defined as, $N_{X} = |X - X^{\prime }|$ and $N_{Y} = |Y - Y^{\prime }|$ represents the assay-associated variations in analyte expression. To ensure robustness, we combine $N_{X}$ and $N_{Y}$ into a single worst-case-scenario random variable $N = \max (N_{X}, N_{y})$. The differential analyte expression between the two samples is given by $Z= |X-Y|$. By construction, *N* and *Z* are ratio-scale variables.

For differential expression *Z* to represent a valid difference in the analyte expression between the two groups—whether statistically significant or not—its effect size should be greater than that of *N*. That is, the effect, or magnitude of variation in the differential analyte expression between the groups should be greater than the magnitude of variation in assay-associated noise. We note that this formulation is distinct from common metrics of effect-size such as Cohen’s *d*^16^. They are typically used to quantify the effect-size of differential analyte expression between the sample groups, particularly in the context of power analysis. Our formulation, on the other hand, aims to assess the *validity* of the differential analyte expression computed from measurements made in presence of assay noise. It, therefore, is fundamental to all downstream statistical analyses including power analysis.

Mathematically we can express the above relation as $\mathscr {M}(Z) \ge \mathscr {M}(N)$, where $\mathscr {M}$ denotes a measure of the effect-size. Re-expressing it as $\log _{10}\frac{\mathscr {M}(Z)}{\mathscr {M}(N)} \ge 0$, we obtain the fidelity condition in terms of the $\log$-based function of relative change: $f(\mathscr {M}(Z),\mathscr {M}(N)) = \log _{10}\frac{\mathscr {M}(Z)}{\mathscr {M}(N)}$, which satisfies the following important properties^17^,

1a $$\begin{aligned} f(\mathscr {M}(Z),\mathscr {M}(N))&= 0 \Leftrightarrow \mathscr {M}(Z)= \mathscr {M}(N), \end{aligned}$$

1b $$\begin{aligned} f(\mathscr {M}(Z),\mathscr {M}(N))&> 0 \Rightarrow \mathscr {M}(Z) > \mathscr {M}(N), \end{aligned}$$

1c $$\begin{aligned} f(\mathscr {M}(Z),\mathscr {M}(N))&< 0 \Rightarrow \mathscr {M}(Z) < \mathscr {M}(N), \end{aligned}$$

1d $$\begin{aligned} f(\alpha \mathscr {M}(Z), \alpha \mathscr {M}(N))&= \alpha f(\mathscr {M}(Z),\mathscr {M}(N)), \forall \alpha > 0, \text {and} \end{aligned}$$

1e $$\begin{aligned} f(\mathscr {M}(Z),\mathscr {M}(N))&\text { is a continuous and monotonically increasing} \nonumber \\&\text { function of } \mathscr {M}(Z) \text { when }\mathscr {M}(N)\text { is fixed}, \end{aligned}$$

As a result, *f* defines a well-behaved general measure of fidelity that is independent of measurement units and increases or decreases based on whether the effect size of *Z* is greater or less than *N*. Since the $\log$ function satisfies the properties $\log (1) = 0$ and $\log (pq) = \log (p)+\log (q),\forall p,q > 0$^18^, we can naturally extend *f* to combine different measures of relative change into a single $\log$-based sum of relative changes. This extension will also satisfy Eq. (1) properties. We next use this extension to define *rxCOV* fidelity metric as a function of first- and second-order measures of effect-size.

**Figure 1:**
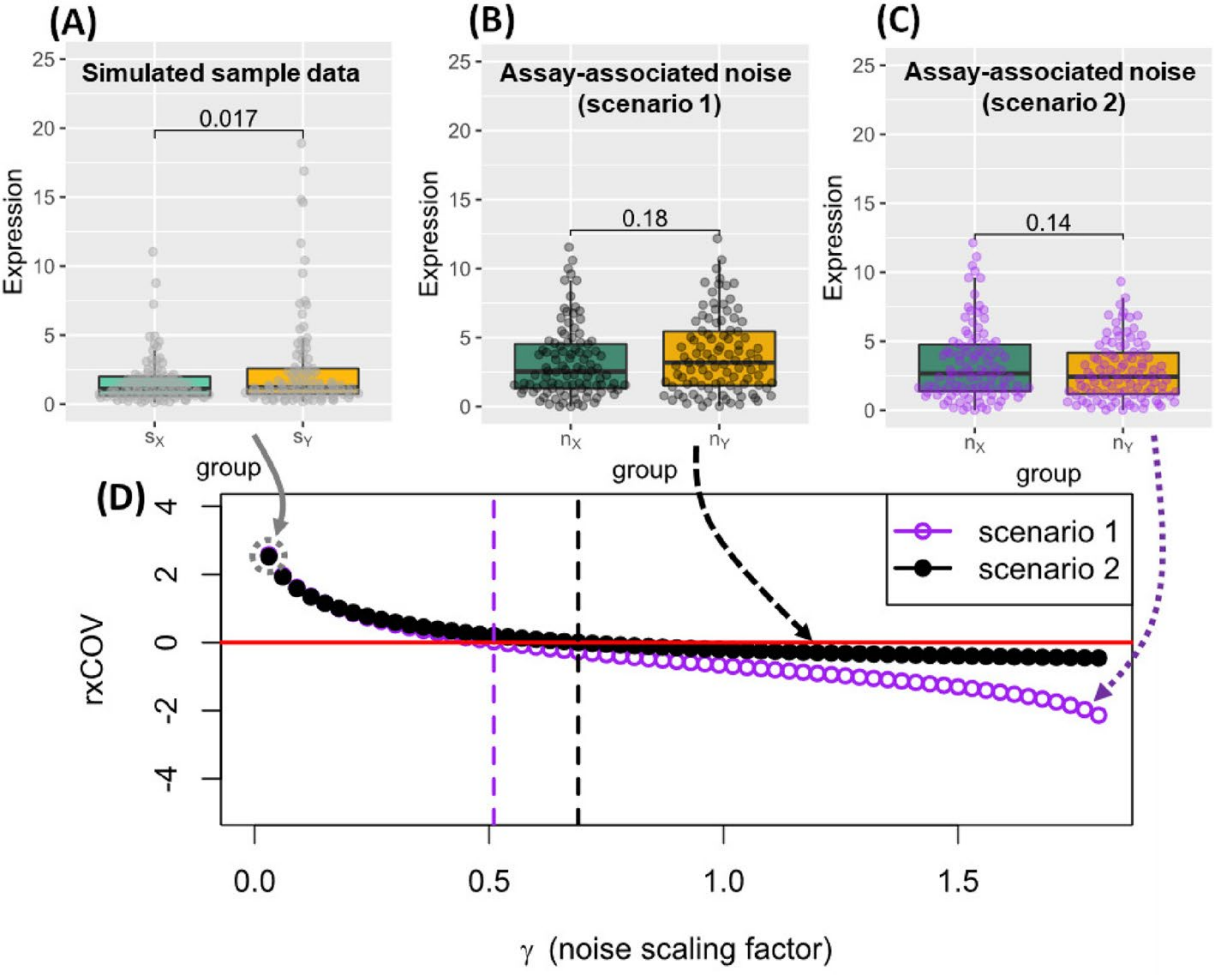
Simulated example of *rx*COV as an objective fidelity metric. (**A**) Analyte data simulating hypothetical cytokine expression in two sample groups $s_{X}$ and $s_{Y}$. The difference in cytokine expression is significant at the 95% confidence level. (**B**) Baseline scenario 1 representing measurement noise associated with assaying each sample of each group. (**C**) Baseline scenario 2 representing a slightly different assay-associated noise distribution than scenario 1. Data $d_{X}$ and $d_{Y}$ are generated by scaling these baseline scenarios by the scaling factor $\gamma$ and adding them to $s_{X}$ and $s_{Y}$ as formulated in Eq. (3). Assay-associated noise in both scenarios is not significant at the 95% confidence level and remains unaffected by the scaling factor $\gamma$. (**D**) *rx*COV plot as a function of $\gamma$ for Scenarios 1 and 2. When $\gamma =0$, $d_{X} = s_{X}$ and $d_{Y} = s_{Y}$, no assay noise is assumed, and the two scenarios coincide. This is indicated by the dashed gray circle. The solid red line corresponds to $rx\text {COV}=0$, the objective fidelity threshold. The purple and black dashed vertical lines correspond to the noise levels at which the *rx*COV plots intersect the solid red line, and indicate the low-fidelity analyte threshold for the two respective scenarios.

#### *rx*COV

We consider mean ($\mu$) and standard deviation ($\sigma$) as two measures $\mathscr {M}_{1}(\cdot )$ and $\mathscr {M}_{2}(\cdot )$ respectively of the effect size of *Z* and *N*. The resulting functions of relative change $f(\mu _{Z},\mu _{N})$ and $f(\sigma _{Z},\sigma _{N})$ respectively quantify the *average* and *dispersion* fidelity of the differential analyte expression between the two sample groups, with respect to the assay-associated noise. As an example, $f(\mu _{Z},\mu _{N}) = 0$ and $f(\sigma _{Z},\sigma _{N}) = 0$ imply that effect-sizes related to mean (first-order) and dispersion (second-order) of differential analyte expression are the same as that of assay-associated noise. As a result, differences in analyte expression between the two groups or conditions cannot be separated from differences arising due to assay-associated variability. Consequently, the analyte has low-fidelity for this specific assay.

Utilizing the additive property of *f*, we combine the first- and second-order fidelity measures of effect size and define our joint fidelity metric *rxCOV* as

2a $$\begin{aligned} rxCOV(Z,N)&= \log _{10}\frac{\mathscr {M}_{1}(Z)}{\mathscr {M}_{1}(N)} + \log _{10}\frac{\mathscr {M}_{2}(Z)}{\mathscr {M}_{2}(N)}, \end{aligned}$$

2b $$\begin{aligned} rxCOV(Z,N)&= \log _{10}\frac{\mu _{Z}}{\mu _{N}} + \log _{10}\frac{\sigma _{Z}}{\sigma _{N}}, \end{aligned}$$

2c $$\begin{aligned} rxCOV(Z,N)&= \log _{10}\frac{(\sigma _{Z}/\mu _{N})}{(\sigma _{N}/\mu _{Z})}. \end{aligned}$$

Equation (2c) has an intuitive interpretation: It is a *standardized* measure of dispersion of differential analyte expression between two sample groups, relative to dispersion in assay associated noise. The standardization of $\sigma _{Z}$ by $\mu _{N}$, and $\sigma _{N}$ by $\mu _{Z}$, ensures that the comparison of relative dispersion is adjusted to the correct scale. It also motivates the name of the fidelity metric *ratio of cross-Coefficient-of-Variations* (*rxCOV*). Here, ‘cross’ refers to standardization of $\sigma _{Z}$ by $\mu _{N}$, and of $\sigma _{N}$ by $\mu _{Z}$. If $rx\text {COV}(Z,N) > 0$, then the range of variation in the differential analyte expression after having been adjusted by average assay-associated noise dominates the variation in assay-associated noise adjusted by the mean differential analyte expression. Consequently, the analyte has high fidelity. If, on the other hand, $rx\text {COV}(Z,N) \le 0$, then the analyte has low fidelity.

We emphasize that Eq. (2c) is easy to implement. As the equation shows, computation of *rx*COV is straightforward. It requires computing four simple statistics: mean and standard deviation of differential analyte expression (*Z*) and assay-associated noise (*N*) measurements. This can be done using any rudimentary statistical software, thereby making *rx*COV an easy-to-use metric for researchers from diverse backgrounds.

**Figure 2:**
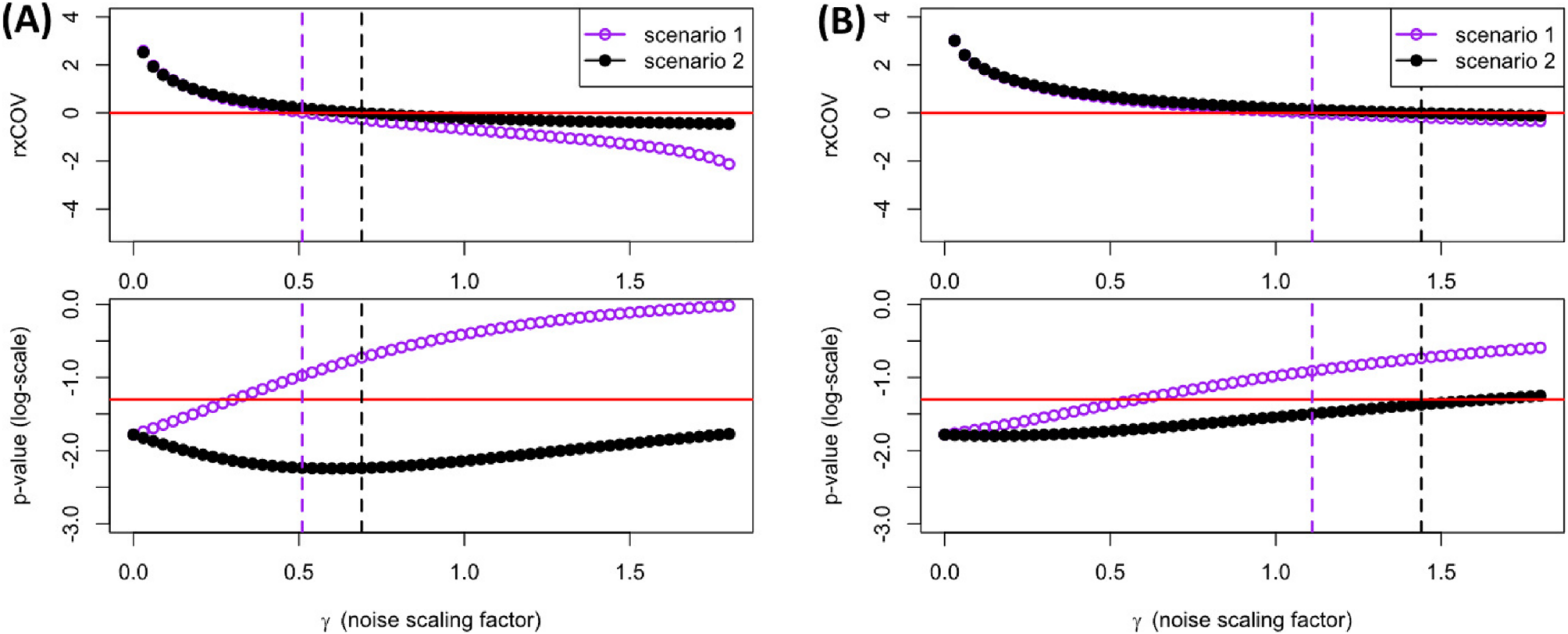
Simulated example of *rx*COV identifying spurious statistical significance and capturing effect of experimental replication. (**A**) Fig. 1D is extended to include the relation between *rx*COV and p-value as a function of $\gamma$ for the two noise scenarios presented in Fig. 1B,C. The solid red line in the p-value plot (bottom panel) indicates p-value of 0.05. The dashed vertical lines corresponding to the two scenarios continue over from the top panel and correspond to the value of $\gamma$ where *rx*COV reaches zero. They establish the noise level beyond which p-value based computational of statistical significance becomes spurious. (**B**) Three replicates of $s_{X}$ and $s_{Y}$ are generated from the same distribution used in Fig. 1A. Corresponding values of $d_{X}$ and $d_{Y}$ are computed and averaged to increase analyte SNR. The rightward shift of the purple and black dashed vertical lines coupled with the convergence of the *rx*COV plots for the two scenarios demonstrates the ability of *rx*COV to capture the increased SNR due to data averaging.

### *rx*COV is a smooth function of its variables

The *rxCOV* metric, given by Eq. (2c), is a function of four variables $\mu _{Z}, \mu _{N}, \sigma _{Z}, \sigma _{N}$, defined on the open region $\mathscr {D} = (0, \infty )^{4} \subset \mathscr {R}^{4}$. Its partial derivative with respect to each of the four variables is a hyperbola, a continuous function over the domain $(0, \infty )$^19^. As a result, *rxCOV* is differentiable over $\mathscr {D}$, which in turn ensures that it is also a continuous function over $\mathscr {D}$^20^. Furthermore, with hyperbola as the partial derivative of first-order for each of the four variables, the existence of partial derivatives of *rxCOV* of any order is guaranteed. Therefore, *rx*COV is not only a continuous function over $\mathscr {D}$, but it is also a smooth function over it. This property ensures that the small changes in $\mu _{Z}, \mu _{N}, \sigma _{Z}, \sigma _{N}$ result in small changes in *rx*COV. Thus, *rx*COV can smoothly track analyte fidelity across the whole range of differential analyte expression.

### *rx*COV is an objective fidelity metric

We constructed a dataset comprising simulated expression of a cytokine in two samples groups, denoted by $s_{X}$ and $s_{Y}$ (Fig. 1A). The expressions are chosen such that the differential expression between the two sets is significant at the $95\%$ confidence level, with a p-value of 0.017. Two scenarios based on two different assay-dependent noise profiles ($n_{X}$ and $n_{Y}$) are considered (Fig. 1B,C). The base noise profiles, however, are not statistically significant at the $95\%$ confidence level. The final dataset for the two scenarios is constructed as follows,

3a $$\begin{aligned} d_{X}&= s_{X} + \gamma n_{X}, \end{aligned}$$

3b $$\begin{aligned} d_{Y}&= s_{Y} + \gamma n_{Y}. \end{aligned}$$

Here $\gamma$ scales the base noise profile to recapitulate a range of assay-dependent noise scenarios, without affecting the relative significance of the noise profiles themselves within each of the two scenarios. The resulting plots of *rx*COV as a function of $\gamma$ for the two scenarios are shown in Fig. 1D. The color coded dashed vertical lines indicate where *rx*COV curve reaches zero. At this zero threshold, the effect-sizes of differential cytokine expression between the two sample groups and that of the the assay associated noise with respect to mean and dispersion are the same and cannot be distinguished from each other. Above this threshold the effect size of the differential expression dominates the noise and the analyte has high fidelity, while below this threshold the converse is true and the noise dominates. Therefore, *rx*COV is an experiment-specific metric that objectively assesses analyte fidelity via the zero-level threshold.

### *rx*COV identifies spurious statistical significance

In Fig. 2A, along with the plot of *rx*COV as a function of $\gamma$ for the two scenarios shown in Fig. 1D, we also plot the p-value corresponding to statistical significance of the differential expression between $d_{X}$ and $d_{Y}$ as a function of $\gamma$. In this plot the solid red line indicates statistical significance at the 95% confidence level.

Scenario 1 reflects a situation where increasing noise level gradually washes out the significance of the data. Here, the differential expression becomes insignificant before *rx*COV reaches 0, as indicated by the purple dashed vertical line. This result indicates that although, the first- and second-order effect sizes of the differential expression between $d_{X}$ and $d_{Y}$ are greater than that of assay-associated noise, they are not large enough to ensure statistical signficance at the 95% confidence level. Once *rx*COV reaches 0, the assay-associated noise dominates and statistical signficance becomes spurious. *rx*COV, therefore, establishes the sufficient condition for the validity of the p-value in assessing statistical significance of the differential analyte expression. Specifically, if $rx\text {COV} \le 0$ then any p-value, whether $\lessgtr 0.05$ or $\le 0.001$ is a noise related artifact and is meaningless.

Scenario 2 illustrates a situation where the differential expression between $d_{X}$ and $d_{Y}$ remains significant beyond the black dashed vertical line, where *rx*COV reaches zero, and the first- and second-order effect size of assay-associated noise begin overwhelming the differential analyte signal. *rx*COV identifies this significance to be spurious. Importantly, it exemplifies how a small stochastic change in the noise distribution from that of scenario 1 can result in wide divergence in p-value behavior of the differential analyte expression, thereby emphasizing the critical importance of a fidelity metric.

**Figure 3:**
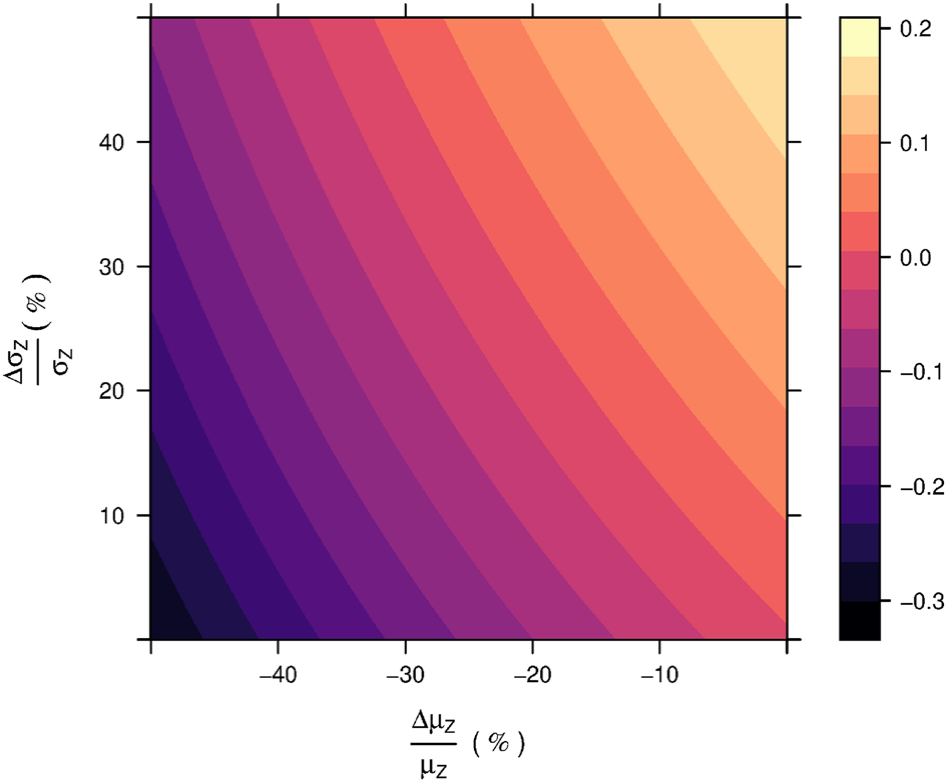
Relative change in *rx*COV under the paired vs. unpaired assumptions. Plot of $\Delta rx\text {COV}$ as a function of relative deviation in mean and standard deviation resulting from differences due to the paired and unpaired assumption. The deviation in relative mean value ranges from 0% through 50%, while that in standard deviation ranges from − 50% through 0%. The colorbar indicates the corresponding deviation in $\Delta rx\text {COV}$.

### *rx*COV and experimental replication

A common approach for improving the fidelity of analyte expression is to average multiple independent replicates. Figure 2B demonstrates that *rx*COV fidelity metric is able to capture the resulting increase in signal to noise ratio via the rightward shift of the purple and black dashed vertical lines corresponding to $rx\text {COV}=0$. In it, the same two scenarios presented in Fig. 2A are considered, but instead of a single measurement, three independent replicates of $d_{X}$ and $d_{Y}$ are generated and averaged. As expected, the *rx*COV and p-value plots corresponding to the two scenarios as a function of $\gamma$ converge towards each other. Furthermore, the values of $\gamma$ at which *rx*COV reaches zero increases, marked by the rightward shift of the dashed vertical lines, and indicates the averaging-dependent increase in noise range over which the signal maintains its fidelity with respect to assay-associated noise. Interestingly, despite the benefit of data averaging, the risk of spurious significance is not completely removed. This is evident in scenario 2, where p-value remains below 0.05 immediately after *rx*COV reaches 0. Therefore, even though averaging ameliorates noise effect, it remains difficult to ascertain if the number of replicates are enough to overcome the noise level of the specific assay. In such a situation, *rx*COV metric can provide the answer: if the *rxCOV* value resulting from using averaged replicates is $>0$, the number of replicates is sufficient. If, on the other hand, it is $\le 0$, then more replicates are required. Importantly, *rx*COV metric can also be used to decide for which analytes data averaging is not needed, particularly in multiplexed scenarios where replication can be limited by cost, sample, and logistical constraints.

### *rx*COV is a fidelity metric for paired and unpaired data

Rewritng the definition of $Z = |X-Y|$ in terms of the lattice identity $Z = |X-Y| = \text {max}(X,Y) - \text {min}(X,Y)$^21^ shows that this definition depends on pairwise max and min operations and assumes paired data. For unpaired data, we redefine *Z* as $Z^{up} = X-Y$, with its mean and standard deviation respectively given by $\mu ^{up}_{Z} = \mu _{X} - \mu _{Y}$ and $\sigma ^{up}_{Z} = \sqrt{\sigma ^{2}_{X} + \sigma ^{2}_{Y}}$. The corresponding measures of effect size, then are $\mathscr {M}_{1}^{up}(Z) = |\mu ^{up}_{Z}|$ and $\mathscr {M}_{2}^{up}(Z) = \sigma ^{up}_{Z}$, and we can use Eq. (2c) to compute analyte fidelity. We note that the first- and second-order measures of effect-size of assay-dependent noise are always paired because, by experimental design, assay-associated expression variability is quantified via repeated measurement of analyte expression of the same sample. The paired or unpaired status of the data itself is determinded by the study and experimental design and is not a matter of preference. Nevertheless, in order to develop a comprehensive understanding of the fidelity metric, we computed the deviation in *rx*COV value for the same data under paired and unpaired assumptions. We first defined difference in the mean and standard deviation via the following relations,

4a $$\begin{aligned} \mu ^{up}_{Z}&= \mu _{Z} + \Delta \mu _{Z}, \Delta \mu _{Z} \le 0, \end{aligned}$$

4b $$\begin{aligned} \sigma ^{up}_{Z}&= \sigma _{Z} + \Delta \sigma _{Z}, \Delta \sigma _{Z} \ge 0 , \end{aligned}$$

with the inequalities $\Delta \mu _{Z} \le 0$ and $\Delta \sigma _{Z} \ge 0$ following directly from the lattice identify representation of *Z*. The relative difference in the *rxCOV* fidelity metric between the paired and unpaired assumption can then be expressed as,

5 $$\begin{aligned} \Delta rxCOV = \log _{10}\left( 1+\frac{\Delta \mu _{Z}}{\mu _{Z}}\right)&+ \log _{10}\left( 1+\frac{\Delta \sigma _{Z}}{\sigma _{Z}}\right) , \nonumber \\&\text {with } \Delta \mu _{Z} \le 0, \Delta \sigma _{Z} \ge 0. \end{aligned}$$

Here, $\frac{\Delta \mu _{Z}}{\mu _{Z}}$ and $\frac{\Delta \sigma _{Z}}{\sigma _{Z}}$ respectively capture the relative difference between the paired and unpaired assumption for $\mu$ and $\sigma$ with respect to the paired assumption. Figure 3 shows that for a broad range of deviation, from $0\%$ through $50\%$, $\Delta rxCOV$ is bounded within the range $(-\, 0.301, 0.2)$, implying that although *rx*COV is sensitive to the paired and unpaired assumption, it remains a relatively stable and bounded metric, for a large range of variation.

**Figure 4:**
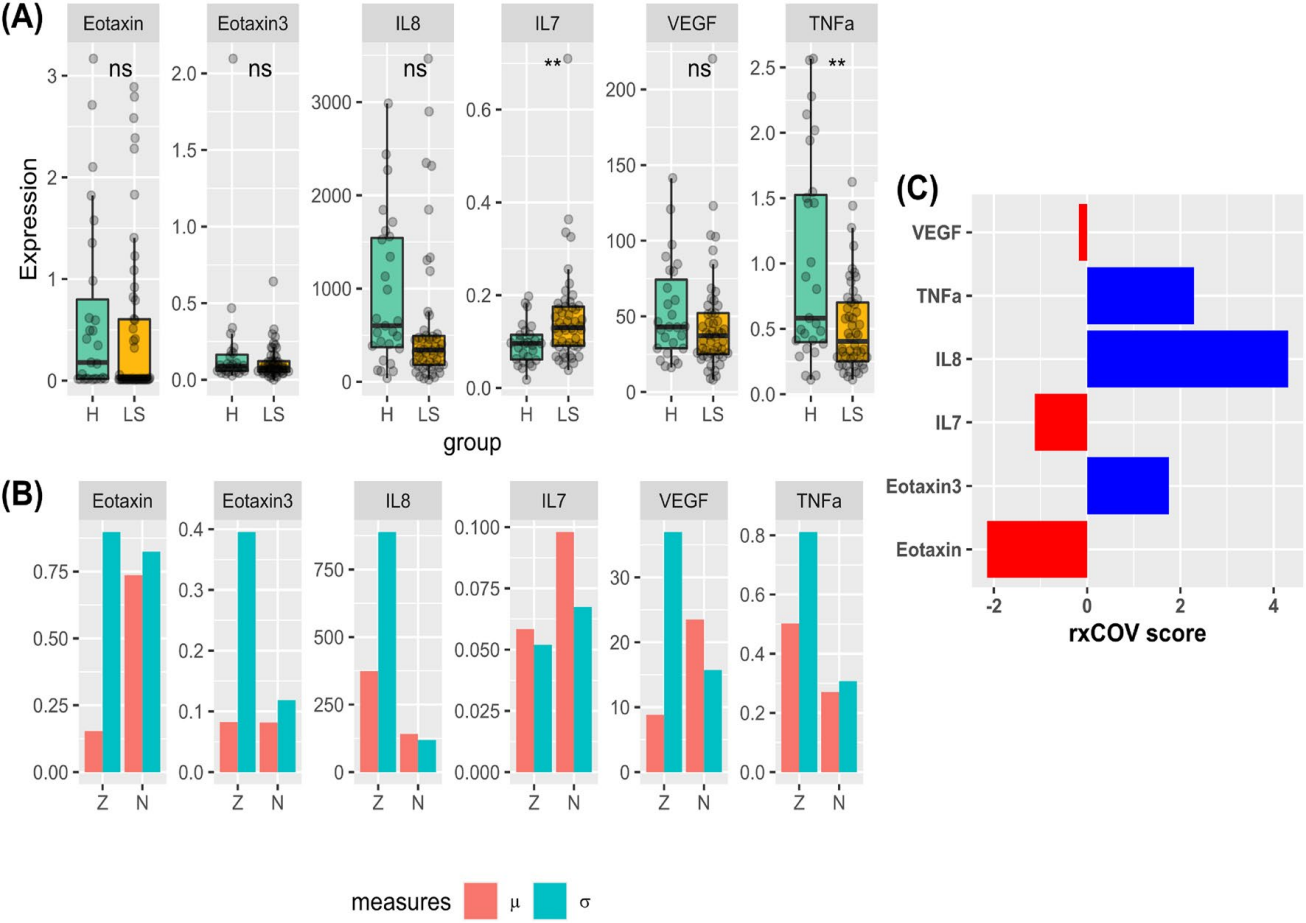
Case study. (**A**) Boxplot representation of multiplexed ELISA expression of six cytokines measured in Lynch Syndrome (LS) patients with healthy (H) patients acting as controls. (**B**) Barplots of the mean and standard deviation of the cytokine differential expressions (*Z*), and their corresponding assay-associated noise (*N*). (**C**) *rx*COV metric computed by substituting the mean and standard deviation of the differential expression, shown in (**B**), for each of the six cytokines . Those with $rx\text {COV} > 0$ are high-fidelity analytes and are shown in blue. Those with $rx\text {COV} \le 0$ are low-fidelity analytes and are shown in red.

### Case study: establishing the fidelity of immune signaling profile in patients with Lynch syndrome

Figure 4A shows a subset of multiplexed ELISA expression data collected from healthy volunteers (H) and patients with Lynch syndrome (LS) for six chemotactic, proinflammatory and immune suppressive cytokines (see “Materials and methods” section). The differential expression of the six cytokines between healthy volunteers and LS patients captures differences in the immune signaling microenvironment of these groups of patients. The figure also indicates the significance of this difference at the 95% confidence level. Figure 4B plots the mean and standard deviation of differential expression (*Z*) and assay-associated noise (*N*) for the same set of cytokines. The figure makes it evident that despite paying careful attention to experimental protocols that help ameliorate the effects of assay-associated noise, for some cytokines assay-associated noise still overwhelms the differential expression. We note that the interplay between signal and noise is sample and assay dependent. The importance of *rx*COV is in its ability to objectively quantitate this sample and assay dependent interplay and establish the fidelity of each individual cytokine. Figure 4C shows that *rx*COV fidelity metric is able to identify high and low fidelity cytokines. Interestingly, although we see that differential expression of both IL7 and TNF$\alpha$ is statistically significant, the *rx*COV fidelity metric shows that for IL7 this significance is spurious ($rx\text {COV} < 0$) and is an assay-associated artifact. On the other hand, differential expression of TNF$\alpha$ is significant ($rx\text {COV} > 0$). Importantly, *rx*COV also indicates where lack of significance might be an artifact. For example, although both Eotaxin and Eotaxin3 are not statistically significant, this lack of significance for Eotaxin could potentially be an experimental artifact ($rx\text {COV} < 0$), while for Eotaxin3 it is not ($rx\text {COV} > 0$). Thus, these results demonstrate the practical way in which *rx*COV metric can guide experimental decisions in real-time.

## Discussion

We have introduced *rx*COV, a simple and easy-to-use quantitative metric for assessing the fidelity of immunoassay based analyte measurements comparing different treatment conditions or sample groups. As shown in Eq. (2c), *rx*COV only requires computation of mean and standard deviation of the differential expression (*Z*) and assay-associated noise (*N*). *Z* is obtained from assay measurements of the samples under study. *N* is also simple to compute and only requires an additional measurement of an aliquot of the same set of samples, as exemplified by the case study. The computations can be easily performed using a low-end computer equipped with a statistical software capable of performing subtraction, and computation of mean and standard deviation of expression values from a sample group. Therefore, *rx*COV is accessible to researchers in diverse research, translational and clinical settings with varying computational expertise. Importantly, unlike other common and important metrics of effect size such as Cohen’s *d* and its variations, that are used to quantify the size of the effect—that is, the magnitude of the differential analyte expression—between sample groups or treatment conditions, *rx*COV assesses the validity of the differential analyte expression in presence of assay noise. Additionally, it establishes a sufficient condition for the validity of p-value and statistical significance associated with the differential analyte expression. Thus, it is fundamental to all downstream analyses and interpretation.

The strength of *rx*COV is its objective threshold of zero that demarcates the boundary between experiment-specific analyte fidelity and the lack of it. From the construction of the metric, it is evident that zero is the correct fidelity threshold because it identifies the point of balance between the differential analyte expression and the noise embedded in the measurement of analyte expression for a specific experiment. For *rx*COV values $>0$, the differential signal is stronger than the noise, while it is the opposite for $rx\text {COV} < 0$. Using the shift in this threshold, we demonstrated the senstivity of *rx*COV to averaging experimental replicates, an important validation of the metric being able to measure analyte fidelity. Importantly, it identifies another use of *rx*COV as an objective criterion in determining the optimal number of replicates needed to ensure signal fidelity of an analyte.

*rx*COV uses mean and standard deviation summary statistics to quantify the effect-size of properly normalized differential analyte expression and its noise (Eq. 2c). However, *rx*COV is not limited to the use of these statistics in two ways. First, if the mean and standard deviation are skewed by the nature of the study data, they can respectively be replaced by median and interquartile range (IQR) that could be more robust to outliers^22^. Second, *rx*COV can be naturally extended to incorporate additional summary statistics, such as entropy, based on the additive property of the function of relative change as shown in Eq. (2a). However, use of additional statistics should be supported by a rationale relevant for the study and the nature of its data. Typically, our use of first- and second-order summary statistics should be sufficient and accurate in most cases.

For computing summary statistics of assay noise within a sample group or treatment condition associated with measurement of analyte expression, *rx*COV requires a single repeat measurement per sample. This modest requirement follows from the observation that variations in repeat measurements of the same sample fundamentally capture stochastic and assay-associated noise, and are independent of the sample itself. Thus, single repeat measurement per sample, for all samples in the study, provides enough stochastic diversity to reliably compute noise summary statistics. The same summary statistics can also be computed using repeat measurements ($>1$) of aliquots drawn from a subset of samples. The optimal choice between these range of options is dependent upon sample constraint specific to the study. Computation of *rx*COV can, therefore, be adapted to the constraints and logistics of individual research studies, thereby suggesting its wide applicability.

As *rx*COV is computed per analyte, it can be easily computed for multiple analytes measured in parallel, for example, in the case of multiplexed immunoassays. We demonstrated this generalizability to a multiplexed setting in the case study presented above. There *rx*COV was used to simultaneously assess the fidelity of all six analytes. Additionally, its pairwise computation for any two—paired or unpaired—treatment conditions or sample groups, can also be easily extended to multiple experimental or sample group comparisons, each implemented pairwise. Importantly though, given its simple formulation, increase in number of analytes and sample group comparisons will not unduly increase the computational complexity of *rx*COV based fidelity analysis as *rx*COV only requires computation of four values—two mean and two standard deviation values—per comparison.

*rx*COV can also be used with other biosensing assays beyond antibody-based immunoassays. As noted in the Introduction, aptamer-based ELAA assays replace antibody based detection with aptamer probes. Nevertheless, like ELISAs, ELAA measurements are usually based on calorimetric, fluorescence, or chemiluminiscence principles, ensuring direct applicability of *rx*COV^8^. Beyond aptamer-based assays, *rx*COV can also be applied to other types of biosensing assays where the measurements are ratio scale variables, that is, analyte expression value of zero is well-defined and biochemically meaningful.

## Conclusion

We have introduced *rxCOV*, a simple, quantitative metric to determine whether the differential expression of analytes between two sample or treatment groups is valid with respect to the assay-associated noise in individual experiments. *rx*COV is applicable when measurements are made using antibody-based immunoassays, aptamer-based biosensing assays, and for assays where the measured quantity is a ratio scale variable with zero-value analyte expression being biochemically meaningful. It is easy to use and requires computation of only mean and standard deviation. Its key strength is the objective threshold of zero that establishes experiment-specific analyte fidelity that also provides a sufficient condition for the validity of the p-value based claim of statistical significance. Since *rx*COV is computed per analyte at a very low computational cost, it can be easily computed for multiple analytes measured in parallel as is the case for multiplexed assays. Additionally, it can also be extended to multiple sample group comparisons, each implemented pairwise. Finally, *rx*COV can help determine the optimal number of experiment-specific replicates required to ensure analyte fidelity. We anticipate that these strengths will make *rx*COV an attractive metric in aiding implementation of robust and reproducible biomedical research studies.

## Materials and methods

### Tissue collection

The data for the case study demonstrating the practical efficacy of *rx*COV, was collected as part of a separate study of patients with Lynch Syndrome (LS), the most common cause of hereditary colorectal cancer. The study (STUDY20010017) was approved by the Institutional Review Board at the University of Pittsburgh, Pittsburgh, PA and adhered to the University guidelines for research involving human subjects. All participants signed informed consent documents. Biopsies of normal-appearing colorectal mucosa were obtained from 16 healthy volunteers and 28 LS patients. All biopsies were obtained during routine colonoscopies performed at University of Pittsburgh Medical Center, Shadyside Hospital, Pittsburgh, PA, USA.

### Materials: chemicals and reagents

All transport and tissue culture media ingredients were purchased from Thermo Fisher Scientific, (Waltham, Massachusetts), including RPMI 1640 (Cat $\#22400105$) , heat inactivated fetal bovine serum (HI-FBS, Cat $\#10082147$) and Antibiotic-Antimycotics (Cat $\#15240062$). Biomarker assays were performed using the Meso Scale Discovery (MSD, Rockville, MD) V-PLEX Human Cytokine 30-Plex Kit (Cat $\#\text {K}15054\text {D}$).

### Multiplexed ELISA on explant cultures

Intact biopsies collected from healthy and LS participants were immediately placed into tubes containing tissue culture media comprised of RPMI 1640, 7.5% HI-FBS and 1% antibiotic-antimycotic. Samples were maintained on ice and transported to the laboratory in a biohazard container. Tissues were immediately weighed and placed into prefilled individual wells of a 24-well tissue culture plate containing 1 mL of complete RPMI (cRPMI;RPMI 1640, 10% HI-FBS, $1\%$ antibiotic/antimycotic) culture medium in a biosafety cabinet and incubated for 24 h at 37 $^{\circ }\text {C}$ with 5% CO2, as described previously^23,24^. Soluble biomarkers released into the supernatant through 24 h of culture were aliquoted and measured using MSD mELISA. Measured biomarkers included a range of chemotactic, immunosuppressive and proinflammatory cytokines used to profile the immune signaling microenvironment of the tissue samples. All assays were performed according to the manufacturer’s instructions. To remove variations due to size of tissue biopsy, we normalized the assayed expression by tissue weight. The mELISA assay was also repeated on a second aliquot from each sample. Differences between these two sets of measurements were used as a quantification of assay-associated experimental variability.

### Software

R programing language implemented within the RStudio integrated development environment was used to perform all analysis. We note that no specialized packages are required to implement the *rx*COV metric itself.

## Data Availability

The simulated and experimental data supporting the development and findings of this work are available from the corresponding author upon reasonable request.
